# Solubility of ammonium metal fluorides in aqueous ethanol mixtures – implications for scandium recovery by antisolvent crystallization†

**DOI:** 10.1039/d2ra07516d

**Published:** 2023-01-05

**Authors:** Edward Michael Peters, Michael Svärd, Kerstin Forsberg

**Affiliations:** a MEAB Chemie Technik GmbH Dennewartstraße 25 52068 Aachen Germany; b KTH Royal Institute of Technology, Department of Chemical Engineering SE-100 44 Stockholm Sweden kerstino@kth.se

## Abstract

The recovery of scandium from waste streams of other mining and metallurgical processing industries is gaining research interest due to the scarcity of scandium-containing ores. Hydrometallurgical techniques such as leaching, solvent extraction and crystallization amongst others have been successfully applied to recover scandium salts from such waste streams. Scandium can be recovered as (NH_4_)_3_ScF_6_ by antisolvent crystallization from NH_4_F strip liquors obtained after solvent extraction. The coextraction of metal impurities such as Fe, Al, Zr and Ti causes contamination of the final solid product. The extent of coprecipitation of ammonium metal fluorides depends on their initial concentration in the strip liquor and their solubility in the NH_4_F–antisolvent mixtures. Here, the solubility of ammonium metal fluorides of Sc, Zr, Fe, Al and Ti is reported separately in 3 mol L^−1^ NH_4_F–ethanol mixtures at 25 °C as well as in a system containing all five solid phases. The solubility of (NH_4_)_3_ZrF_7_ is slightly higher than that of (NH_4_)_3_ScF_6_, while the solubilities of (NH_4_)_3_FeF_6_ and (NH_4_)_3_AlF_6_ are significantly lower in comparison to (NH_4_)_3_ScF_6_. The solubility of (NH_4_)_2_TiF_6_ is 1–2 orders of magnitude higher than those of other ammonium metal fluorides. When a mixture of ammonium metal fluoride salts is dissolved in the same 3 mol L^−1^ NH_4_F–ethanol mixture as for the individual salts, the resultant solubility of the ammonium metal fluorides of Sc, Zr and Fe decreases significantly, while the resultant solubility of ammonium aluminum hexafluoride increases. This is likely due to changes in solution speciation with increased NH_4_F concentration and ionic strength.

## Abstract 2

The solubility of ammonium metal fluorides is evaluated in NH_4_F–ethanol mixtures and the implications for antisolvent crystallization of (NH_4_)_3_ScF_6_ are discussed.

## Graphical abstract

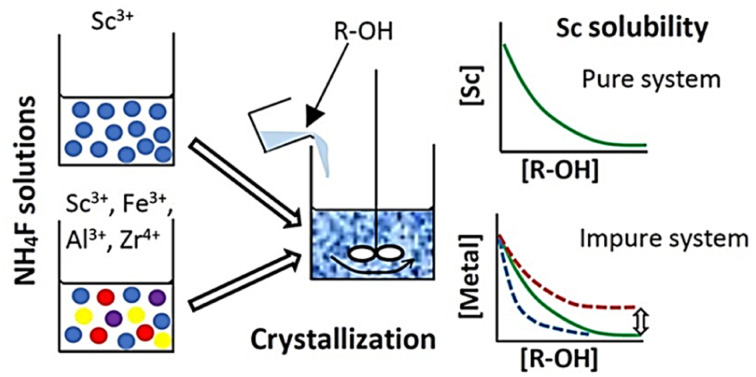

## Introduction

The recovery of scandium from waste streams of other mining and metallurgical industries has gained enormous interest and is essential to meet the increasing global scandium demand. Scandium is usually present in these streams in quantities of economic value and examples of such streams are the bauxite residue from the alumina industry, titanium dioxide acidic waste, slags from iron smelting, as well as uranium and tin processing waste streams.^1–4^ Several techniques have been explored to recover scandium from such streams and these include hydrometallurgical techniques such as leaching, solvent extraction and crystallization.^1,2,5–7^ In recent research, it was reported that antisolvent crystallization is a viable method for recovering scandium as ammonium scandium hexafluoride, (NH_4_)_3_ScF_6_, from NH_4_F strip liquors obtained after acidic leaching and solvent extraction. The percentage recovery of (NH_4_)_3_ScF_6_ from the strip liquors was reported to be *ca.* 99% at an ethanol to strip liquor volumetric ratio of *ca.* 0.8, and the purity of the solid product approached 99%.^8,9^

During the leaching and solvent extraction stages, some metals such as Fe, Al, Ti, Zr, V, U and Th are usually coextracted and end up in the NH_4_F strip liquor, although a high degree of selectivity can be achieved in the solvent extraction stage. These metals contaminate the solid product obtained during crystallization and it was observed that they are usually present in the solid product in proportions that reflect their relative abundances in the strip liquor.^10^ The commercial use of scandium products in specialized applications such as solid oxide fuel cells, solid oxide electrolyzer cells and 3D printing often requires very high purities >99% Sc_2_O_3_ and/or ScF_3_. Increasing the final product purity to such levels requires knowledge of the solubility of species likely to contaminate the product during antisolvent crystallization, which could facilitate the development of an antisolvent crystallization strategy, such as fractional crystallization, to further purify the product. Therefore, it is necessary to investigate the solubility of ammonium metal fluorides of Sc and major impurity metals such as Fe, Al, Zr and Ti in solvent mixtures typical of the antisolvent crystallization process. In a recent study, the solubility of (NH_4_)_3_ScF_6_ has been investigated at 25 °C in NH_4_F solutions of concentration 2–5 mol L^−1^ dosed with alcohols namely methanol, ethanol, 2-propanol, and 1,3-propane-diol as well as in pure NH_4_F solutions of concentration 0.1–12.2 mol L^−1^.^9,11^ The solubility of (NH_4_)_3_ScF_6_ in HF solutions of concentrations up to 24 mol L^−1^ at 30 and 50 °C is also reported in literature, from which the interpolated solubility value at 3 mol L^−1^ HF and 25 °C is about 3.6 g Sc per L.^12^

The phases of the impurity metals that are likely to co-crystallize include (NH_4_)_3_FeF_6_, (NH_4_)_3_AlF_6_, (NH_4_)_3_ZrF_7_ and (NH_4_)_2_TiF_6_ or (NH_4_)_3_TiF_7_. Such ammonium metal fluorides including the Sc phase have been previously reported to exhibit almost similar XRD patterns when crystallized as a mixture, even from solutions that contained higher quantities of Zr, making their identification and quantification challenging.^10^ The heptafluoride phases of Zr^13,14^ and Ti^15,16^ were reported to be the stable phases under ambient conditions and thermal decomposition of these phases to the hexafluorides was observed to occur at 297 °C and 107 °C, respectively. The solubility of (NH_4_)_2_TiF_6_ in water at 25 °C is reported to be 260 g L^−1^ and that of (NH_4_)_3_ZrF_7_ is reported over a temperature range of 0–104 °C to be in the range *ca.* 0.4–1.2 mol L^−1^ (*ca.* 111–334 g L^−1^).^17^ The solubility of (NH_4_)_3_FeF_6_ was observed to increase with increasing HF concentration (≤20 g L^−1^) and temperature (0–60 °C) at constant NH_4_HF_2_ concentration and decrease upon increasing the NH_4_HF_2_ concentration (50–150 g L^−1^).^18^ The increase in solubility with increased HF concentration could be due to increased complex formation between Fe^3+^ and F^−^ anions, while the increase in NH_4_^+^ concentration introduces a common ion which promotes crystallization of (NH_4_)_3_FeF_6_. The solubility of (NH_4_)_3_AlF_6_ and NH_4_AlF_4_ in water at 25 °C are reported as 8 and 0.5 g L^−1^, respectively.^19^ (NH_4_)_3_FeF_6_ and (NH_4_)_3_AlF_6_ also undergo thermal decomposition to their respective tetrafluoride phases at *ca.* 140 and 170 °C, respectively.^20^

It has been reported that (NH_4_)_3_ScF_6_ is the stable phase and can transform into other phases such as (NH_4_)_5_Sc_3_F_14_ and NH_4_ScF_4_ at different fluoride concentrations (0.1–7 mol L^−1^) and temperatures (18 and 90 °C), with the tetrafluoride phase being the stable phase in pure water.^21,22^ Similar phase transformations were also observed in NH_4_F media at fluoride concentrations below 0.8 mol L^−1^ and beyond this concentration, no phase transformation was observed.^22^ Thermodynamic modelling of a Sc–F system revealed that higher order ScF complexes become more stable with increased fluoride concentration, and are also stable at alkaline pH values compared to lower order ScF complexes, while that of higher order ScOH complexes increase with increasing pH.^8^ The stability of complexes is expressed by their stability or formation constants.

Solution speciation plays a significant role in determining which phases are likely to crystallize under specific conditions. In the presence of other metal ions and other solvents such as ethanol, the solution speciation can be significantly altered since there is competition for ligands amongst the metal ions and there is high possibility of ethanol–cation, ethanol–ligand and ethanol–water interactions which adds to the complexity of the system speciation. This can significantly alter the solubility of ammonium metal fluorides. As far as we know, there is no data in literature on the solubility of ammonium metal fluorides in NH_4_F–ethanol mixtures except the data published for (NH_4_)_3_ScF_6_ in such systems.^11^ For this reason, the solubility is determined herein for ammonium metal fluorides of Zr, Fe, and Al separately in 3 mol L^−1^ NH_4_F–ethanol mixtures at 25 °C and compared with the published data for (NH_4_)_3_ScF_6_.^11^ The solubility is also determined in a system containing a mixture of all five salts including the Ti phase in 3 mol L^−1^ NH_4_F–ethanol mixtures at 25 °C to investigate the extent to which the solubility of these compounds is influenced by the presence of other metal salts in solution.

## Methodology

Ammonium scandium fluoride was synthesized by reacting near-saturated solutions of Sc_2_(SO_4_)_3_ (prepared using >99.9 wt% Sc_2_(SO_4_)_3_) and NH_4_F (prepared using >98 wt% NH_4_F) and details of the procedure are published in literature.^11^ Fe(NO_3_)_3_·9H_2_O of purity >98 wt%, ZrF_4_ of purity >98 wt%, aluminum isopropoxide (Al[OCH(CH_3_)_2_]_3_) of purity >98 wt% and TiF_4_ of purity >98 wt% were used to prepare near-saturated solutions of Fe(NO)_3_, ZrF_4_, Al[OCH(CH_3_)_2_]_3_ and *ca.* 30 wt% TiF_4_ in 6 mol L^−1^ hydrofluoric (HF) acid. These solutions were reacted with saturated solutions of NH_4_F to synthesize the corresponding ammonium metal fluorides, and the solids obtained were washed with *ca.* 10 mL of ethanol, dried under ambient conditions for at least 48 hours, and analyzed by powder X-ray diffraction (XRD) (SIEMENS™ D5000) to determine the phase of the solid and by inductively coupled plasma optical emission spectroscopy (ICP-OES) (ThermoScientific iCAP™ 7400) to determine the purity. For powder XRD measurements, the solid sample was pulverized in a mortar and carefully spread over the sample holder and compacted to maintain a flat surface in order to minimize noise generation. The XRD analysis was conducted using an X-ray source of Cu Kα radiation (*λ* = 1.5406 Å). For ICP-OES analysis, a known weight of the solid sample was first dissolved in distilled water and further diluted in a 3.45% v/v HNO_3_ acid matrix. Six calibration standards ranging between 0.01 and 50 mg L^−1^ were prepared from commercial standards containing 1000 mg L^−1^ of the metal in a HNO_3_ acid matrix. The solubility was determined in quaternary systems of H_2_O–NH_4_F–ethanol–(NH_4_)_*x*_Me^*y*+^F_(*x*+*y*)_, where Me is the metal of concern and also in octonary systems of H_2_O–NH_4_F–ethanol and all 5 ammonium metal fluorides.

### Quaternary system

A 3 mol L^−1^ NH_4_F solution was prepared, and ethanol was added to 20 mL of this solution to attain ethanol concentrations in the range 0.5–9 mol L^−1^. About 1 g of the synthesized solid phase (except the Ti phase) was added to the NH_4_F–ethanol mixtures and the suspension was maintained at 25 °C for 24 hours under agitation at 250 rpm using magnetic stirrers. Temperature control was achieved by means of a thermal water bath equipped with a PT100 temperature sensor with an accuracy of ±0.01 °C. A Traceable® temperature sensor was used to verify the suspension temperatures in the experimental containers with temperature variations amongst the containers not exceeding ±0.04 °C. At the end of the experiments, supernatant samples were collected by means of a syringe fitted with a 0.22 μm PVDF membrane filter. The suspensions were then vacuum filtered, and the solids were dried under ambient conditions and analyzed by powder XRD to check if any phase transformation had occurred. The solubility of each phase for each experimental condition in terms of total metal concentration was determined by analyzing the supernatant samples using ICP-OES after diluting the sample in a 3.45% v/v HNO_3_ acid matrix. The densities of the supernatant samples were also determined by measuring their volumes and weights during dilution. Experiments were conducted in duplicate to assess the reproducibility.

### Octonary system

Four experiments were conducted for each experimental condition, and these were grouped into two sets of duplicates. The first set of duplicate experiments was conducted using 5 g of 3 mol L^−1^ NH_4_F solution and the second set was conducted using 10 g of 3 mol L^−1^ NH_4_F solution. Ethanol was added to the NH_4_F solutions to attain concentrations in the range 0.5–2 mol L^−1^. The procedure was similar to the one described for the quaternary system except that 1–2 g of each of the phases of Sc, Fe, Al and Zr and about 4–6 g of the Ti phase were added to each experimental container to assess the effect of other metal salts on the solubility of each of the phases. The suspensions were agitated at 250 rpm over 24 hours and temperature variations not exceeding ±0.1 °C were detected amongst the containers. It has been shown in previous research that equilibrium is attained after 5 hours for the Sc phase^11^ and after 3–4 hours for ammonium metal fluorides of Ti and Nb in the Ti–Nb–NH_4_F–HF aqueous system.^17^

Comparison of published solubility data for (NH_4_)_3_ScF_6_ obtained from the supersaturated state over 72 hours,^9^ and from the undersaturated state^11^ over 24 hours shows negligible discrepancies. Sampling and analysis were conducted as described for the quaternary system. Since the system contained a mixture of salts, calculations were conducted after obtaining the solubility data to determine if the quantity of the Ti phase added was in excess, to ascertain that it did not dissolve completely. The solids added were determined to be in excess of the solubility of the Ti phase in these solution mixtures and was *ca.* 35 ± 5 wt% for the experiments conducted with 0.5 mol L^−1^ ethanol, which is higher than the reported solubility of (NH_4_)_2_TiF_6_ in water (260 g L^−1^).^17^

## Results and discussion

### Phase determination

**Fig. 1:**
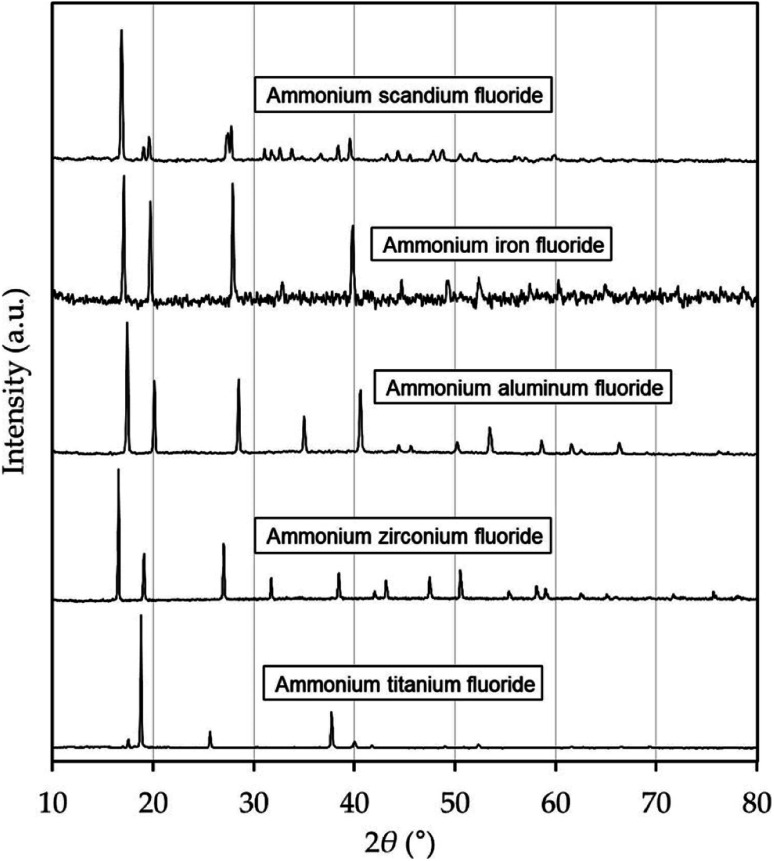
XRD diffractograms of synthesized phases. These were matched with reference patterns obtained from the Powder Diffraction File Database (PDF-2 2021).

The synthesized phases were analyzed by powder XRD and verified to be (NH_4_)_3_ScF_6_ of monoclinic structure, (NH_4_)_3_FeF_6_, (NH_4_)_3_AlF_6_, (NH_4_)_3_ZrF_7_ of cubic structure, and (NH_4_)_2_TiF_6_ of hexagonal structure, respectively. The XRD diffractograms are shown in Fig. 1, and the patterns with overlaid peak positions of reference patterns are given in ESI.† For the Ti Phase, the pattern of (NH_4_)_2_TiF_6_ matched the peak positions almost perfectly, although the peak intensities of the two patterns are not proportionate. It can also be noted that the reference patterns of (NH_4_)_3_TiF_6_ and (NH_4_)_3_TiF_7_ do not match the peaks in the obtained diffractogram, despite the fact that the heptafluoride phase has been reported to transform into the hexafluoride at 107 °C.^15,16^ For this reason, the solubility of the Ti phase is presented as g (NH_4_)_2_TiF_6_ per kg solution. The purity of the solid phases as determined by ICP-OES were 99.96 wt% for (NH_4_)_3_ScF_6_ and >99.9 wt% for the other ammonium metal fluorides. The accuracy in purity determination was within ±0.03 wt%.

### Solubility

#### Quaternary system

**Fig. 2:**
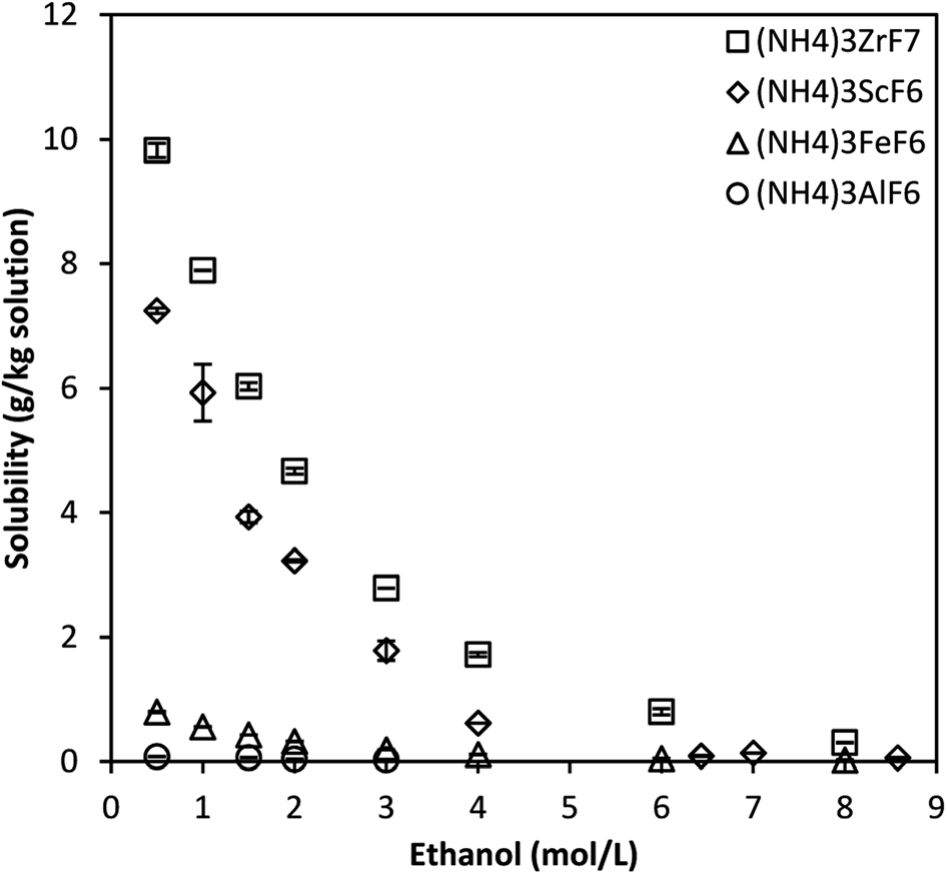
Solubility of ammonium metal fluorides in 3 mol L^−1^ NH_4_F–ethanol mixtures at 25 °C for the quaternary system. Data for the Sc phase is obtained from literature.^11^ Error bars denote the standard deviations from the mean of 2 experimental repeats.

Fig. 2 shows the solubility of ammonium metal fluorides of Zr, Sc, Fe, and Al in 3 mol L^−1^ NH_4_F solutions containing ethanol in the concentration range 0.5–9 mol L^−1^ at 25 °C. This represents the solubility of each salt in the quaternary system consisting of the individual salt, water, NH_4_F and ethanol. It should be noted that the solubility is presented as g of the solid phase per kg total solution (NH_4_F solution + ethanol), while the concentration of NH_4_F given in mol L^−1^ represents that of the fresh solution prepared (before adding ethanol), and the concentration of ethanol is given in mol L^−1^ on a total solution basis (NH_4_F solution + ethanol).

The solubility of (NH_4_)_3_ScF_6_ and (NH_4_)_3_ZrF_7_ in 3 mol L^−1^ NH_4_F solution containing ethanol at a concentration of 0.5 mol L^−1^ total solution is about 10 and 7 g kg^−1^ solution, respectively, while that of (NH_4_)_3_FeF_6_ and (NH_4_)_3_AlF_6_ are *ca.* 0.8 and 0.1 g kg^−1^ solution, respectively. The solubility of all phases decreases asymptotically with increased ethanol concentration, which correlates to the reduction in the effective dielectric constant of the solvent mixture as the ethanol concentration increases.^11,23^ This promotes ion pairing and the crystallization of the respective solid phases of these compounds. Solvents of higher dielectric constant promote complete dissociation in solution. The calculated estimates of the effective dielectric constants of 3 mol L^−1^ NH_4_F solutions dosed with ethanol to attain ethanol concentrations in the range 0.5–9 mol L^−1^ on a total solution basis at 25 °C are published in literature.^11^ In other terms, the solubility of the phases decreases with increase in the concentration of a solvent (ethanol), in which the phases are almost insoluble.

The solubility of the phases decreases in the order Zr^4+^, Sc^3+^, Fe^3+^ and Al^3+^ which correlates to the increase in charge density of the metal ion for the trivalent metals. The calculated charge densities are presented in the ESI.† However, Zr^4+^ has a higher charge density than Sc^3+^, yet the solubility of the Zr phase is higher than that of the Sc phase and this is because the solubility of a compound depends mainly on the energy required to break bonds and is therefore not always correlated to the charge density of the metal ion. The solubility data is presented in Table 1 as averages of two experimental repeats together with their standard deviations. There was no phase transformation detected by powder XRD.

**Table 1:** Solubility data of ammonium metal fluorides of Zr, Fe and Al in 3 mol L^−1^ NH_4_F–ethanol mixtures at 25 °C for the quaternary system. The solubility data of (NH_4_)_3_ScF_6_ is reported in literature^11^

[Ethanol] (mol L^−1^)	Solubility g (NH_4_)_3_ZrF_7_ per kg solution	Solubility g (NH_4_)_3_FeF_6_ per kg solution	Solubility g (NH_4_)_3_AlF_6_ per kg solution
0.5	9.8 ± 0.115	0.80 ± 0.010	0.079 ± 0.003
1.0	7.890 ± 0.003	0.556 ± 0.005	—
1.5	6.03 ± 0.059	0.427 ± 0.002	0.062 ± 0.003
2.0	4.67 ± 0.048	0.321 ± 0.004	0.038 ± 0.003
3.0	2.788 ± 0.001	0.189 ± 0.000	0.028 ± 0.004
4.0	1.72 ± 0.035	0.112 ± 0.004	—
6.0	0.80 ± 0.050	0.047 ± 0.006	—
8.0	0.303 ± 0.004	0.025 ± 0.003	—

#### Octonary system

**Fig. 3:**
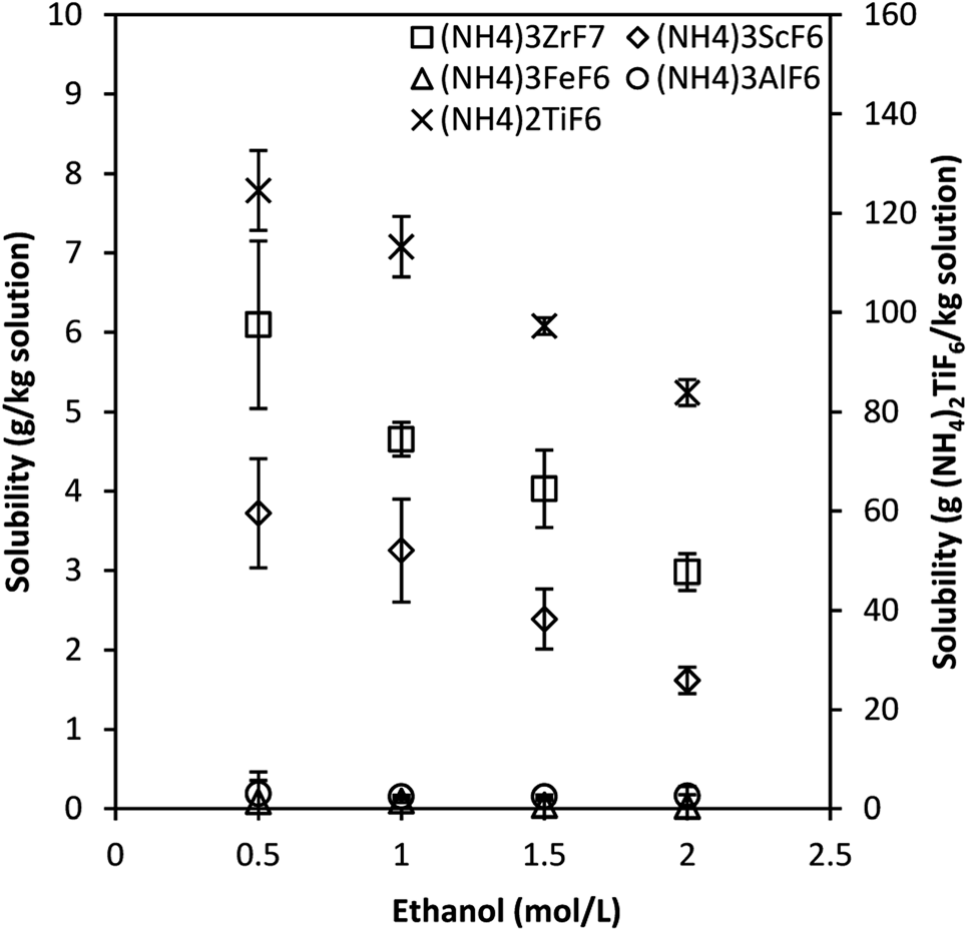
Solubility of ammonium metal fluorides in 3 mol L^−1^ NH_4_F–ethanol mixtures at 25 °C for the octonary system. Error bars denote the standard deviations from the mean of 3–4 experimental repeats.

Fig. 3 shows the solubility, determined in the octonary system, of ammonium metal fluorides of Sc, Fe, Al, Ti and Zr in 3 mol L^−1^ NH_4_F solution containing ethanol in the concentration range 0.5–2 mol L^−1^ at 25 °C. This data considers the effect of other metal salt species on the solubility of the individual salts.

A similar trend in which the solubility of the phases decreases with increased ethanol concentration is observed. The solubility of (NH_4_)_2_TiF_6_ is much higher, about 1–2 orders of magnitude higher than that of other ammonium metal fluorides. This is attributed to the fact that titanium rarely exists as Ti^4+^ in solution and tends to form the stable titanyl complex, TiO^2+^, in solution.^24^

Table 2 shows the solubility data of these phases in the octonary system containing all five salts in a NH_4_F–H_2_O–ethanol system at 25 °C. The data is presented as averages of 3–4 values with their associated standard deviations.

**Table 2:** Solubility data of ammonium metal fluorides of Sc, Zr, Fe, Ti and Al in NH_4_F–ethanol mixtures at 25 °C for the octonary system. The initial NH_4_F concentration is 3 mol L^−1^. The equivalent equilibrium NH_4_F concentration is the sum of the initial 3 mol L^−1^ and the molar quantity of NH_4_^+^ ions that dissolved from each salt and equilibrated with the solid

[Ethanol] (mol L^−1^)	Equivalent equilibrium [NH_4_F] (mol L^−1^)	Solubility g (NH_4_)_3_ZrF_7_ per kg solution	Solubility g (NH_4_)_3_ScF_6_ per kg solution	Solubility g (NH_4_)_3_FeF_6_ per kg solution	Solubility g (NH_4_)_3_AlF_6_ per kg solution	Solubility g (NH_4_)_2_TiF_6_ per kg solution
0.5	4.38	6 ± 1.052	3.7 ± 0.689	0.09 ± 0.082	0.189 ± 0.009	125 ± 8.1
1.0	4.24	4.7 ± 0.213	3.3 ± 0.647	0.10 ± 0.056	0.15 ± 0.020	113 ± 6.1
1.5	4.06	4.0 ± 0.489	2.4 ± 0.379	0.05 ± 0.021	0.15 ± 0.018	97 ± 1.7
2.0	3.91	2.98 ± 0.084	1.6 ± 0.167	0.034 ± 0.003	0.16 ± 0.058	84 ± 2.6

The molar quantity of NH_4_F ions increased due to solubilization of both NH_4_^+^ and F^−^ ions contained in the salts. The NH_4_F concentration values presented in Table 2 are the sum of the initial 3 mol L^−1^ NH_4_F and the equivalent molar quantity of NH_4_^+^ ions that dissolved from all 5 salts and equilibrated with the solid mixture. The term ‘equivalent equilibrium’ is used since the molar quantity of NH_4_F that dissolved was computed as the sum of the molar quantities of NH_4_^+^ ions released by each salt. However, an excess molar quantity of F^−^ ions were released by each salt since the molar ratio of fluoride to ammonium ion concentration is 2.33 for (NH_4_)_3_ZrF_7_, 2 for (NH_4_)_3_ScF_6_, (NH_4_)_3_FeF_6_, (NH_4_)_3_AlF_6_ and 3 for (NH_4_)_2_TiF_6_.

**Fig. 4:**
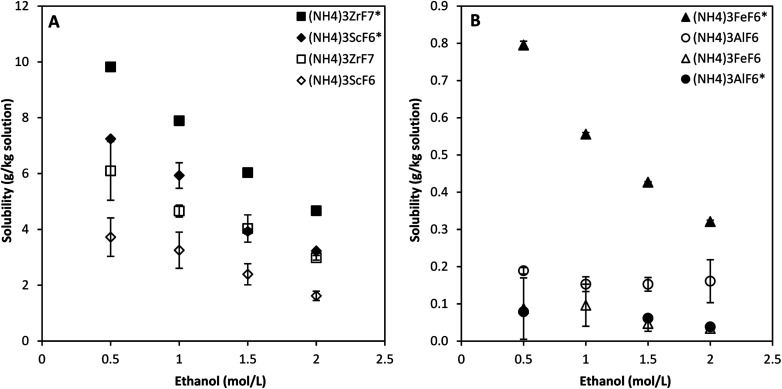
Comparison of the solubility of ammonium metal fluorides in the quaternary (shown by solid markers and asterisks) and octonary (shown by hollow markers) systems. (A) – (NH_4_)_3_ScF_6_ and (NH_4_)_3_ZrF_7_ and (B) – (NH_4_)_3_FeF_6_ and (NH_4_)_3_AlF_6_. Error bars denote the standard deviations from the mean of 2–4 experimental repeats.

Fig. 4 compares the solubility of ammonium metal fluorides of Sc, Zr, Fe and Al determined in the quaternary and octonary systems at 25 °C. The initial NH_4_F concentration was 3 mol L^−1^ in all experiments.

It is observed that the solubility of (NH_4_)_3_ZrF_7_, (NH_4_)_3_ScF_6_ and (NH_4_)_3_FeF_6_ is significantly reduced, by about 37%, 46% and 88%, respectively, for the octonary system compared with the single-salt systems. Furthermore, an increase in the solubility of (NH_4_)_3_AlF_6_ by about 65% was observed in the octonary system. The total equivalent concentration of NH_4_F is higher in the octonary system compared to the single-salt systems (see Table 2), which likely reduces the solubility of species due to the common ion effect, but this does not explain the increase in the solubility of the Al phase.

The changes in solubility are also partly due to changes in solution speciation since the ionic strength increases, and the presence of five metal ions implies that there is competition amongst the ions for ligands in solution. The stability of metal–ligand complexes is expressed in terms of stability or formation constants and the presence of ethanol complicates the system speciation. The stability constants of relevant complexes in the presence of ethanol could not be found in literature.

The XRD diffractogram of the solid mixture after the solubility experiments in the octonary system is presented in the ESI.† The XRD pattern contains multiple peaks on specific 2*θ* angles which are almost matched with the reference patterns of the five ammonium metal fluorides. It can therefore be reasonably assumed that no phase transformations occurred for any of the solid phases under the experimental conditions in this study.

The remarkable changes in the solubility of the ammonium metal fluorides observed in the octonary system compared with the single-metal systems emphasize that the supersaturation experienced by each phase in a system containing a mixture of salts should not be evaluated using solubility data reported in terms of total metal dissolved in pure systems. It illustrates the importance of expressing the supersaturation in terms of the true driving force considering the chemical speciation, which unfortunately is challenging in mixed solvent systems. For instance, a strip liquor that contains 0.02 g (NH_4_)_3_FeF_6_ could be supersaturated with respect to the Fe phase in the octonary system at ethanol concentration ≥3 mol L^−1^ (by extrapolation of the solubility data in Table 2), while it remains undersaturated in the quaternary system at ethanol concentration of 8 mol L^−1^ (see Table 1). The supersaturation experienced by each solid phase can be expressed in terms of the initial total concentration of the metal of that phase and the solubility in terms of total metal concentration of the phase in the system considered. In the typical strip liquors with Sc concentration of *ca.* 2.5 g kg^−1^ solution as presented in published studies,^9,10^ the Sc phase attains supersaturation at an ethanol concentration of about 1.5 mol L^−1^ and the impurity phases become supersaturated at different stages as the ethanol concentration is increased. This means that the purity of the solid phase can be improved by operating at low ethanol concentration, but at the expense of the yield and by conducting stage-wise crystallization. Considering process economy, it would be preferable to maximize the yield given that the purity of the solid is within desired specifications.

## Conclusions

The solubility of ammonium metal fluorides has been determined at 25 °C in quaternary systems containing a single solid phase, water, NH_4_F and ethanol, as well as in octonary systems containing all five salts, water, NH_4_F and ethanol. The data shows that the solubility of ammonium metal fluorides in the quaternary systems decreases asymptotically with increase in ethanol concentration and occurs in the cationic order Zr^4+^, Sc^3+^, Fe^3+^ and Al^3+^. This corresponds to increase in charge density for the trivalent cations while the tetravalent cation (Zr^4+^) deviates from this trend. The presence of other metal salts in the octonary system has a significant effect on the amount of ammonium metal fluorides dissolved. Compared with the respective single-metal systems, the solubility of the ammonium metal fluorides of zirconium, scandium and iron decrease remarkably in the octonary system, while that of (NH_4_)_3_AlF_6_ increases significantly. The changes in solubility are attributed partly to changes in solution speciation as the solution ionic strength increases, and partly to the common ion effect caused by the increase in NH_4_F concentration in the octonary system. In the mixed salt system, there is increase in competition for ligands amongst the metal ions, which is further complicated by the presence of ethanol in the system. The solubility of (NH_4_)_2_TiF_6_ in the octonary system was determined to be 1–2 orders of magnitude higher than that of other phases, most likely due to the formation of the stable TiO^2+^ ion.

## Author contributions

E. M. Peters: conceptualization, methodology, validation, formal analysis, investigation, writing original draft and editing. M. Svärd: conceptualization, writing – review and editing, supervision. K. Forsberg: conceptualization, resources, writing – review and editing, supervision, project administration, funding acquisition.

## Conflicts of interest

There are no conflicts to declare.

## Supplementary Material

RA-013-D2RA07516D-s001
