# Supplementary material for: Solubility of ammonium metal fluorides in aqueous ethanol mixtures – implications for scandium recovery by antisolvent crystallization
Source: RSC Adv. 2023 Jan 5;13(2):1288–94. doi: 10.1039/d2ra07516d (PMC9813942; doi:10.1039/d2ra07516d)
Supplement: RA-013-D2RA07516D-s001 [file RA-013-D2RA07516D-s001.pdf]

Electronic Supplementary Material (ESI) for RSC Advances.
This journal is © The Royal Society of Chemistry 2022

Supplementary Information

I. Phase determination of synthesized solids before and after solubility tests

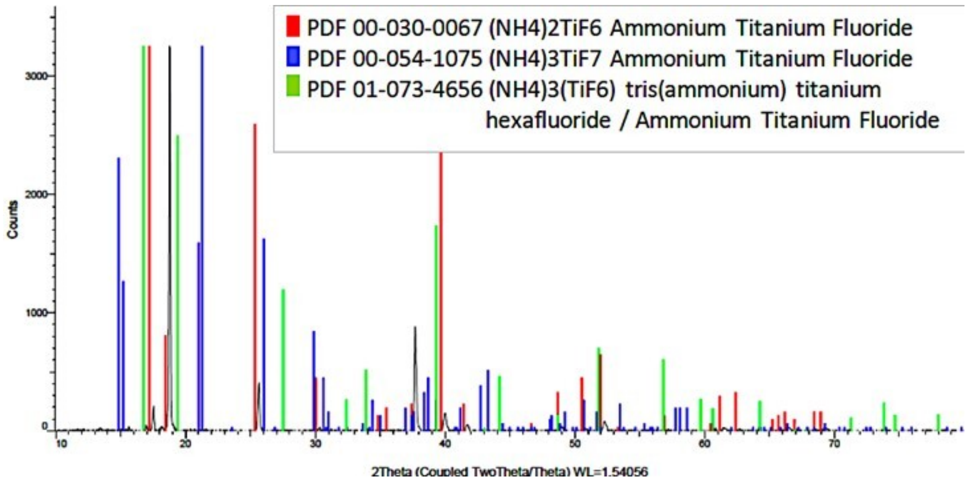

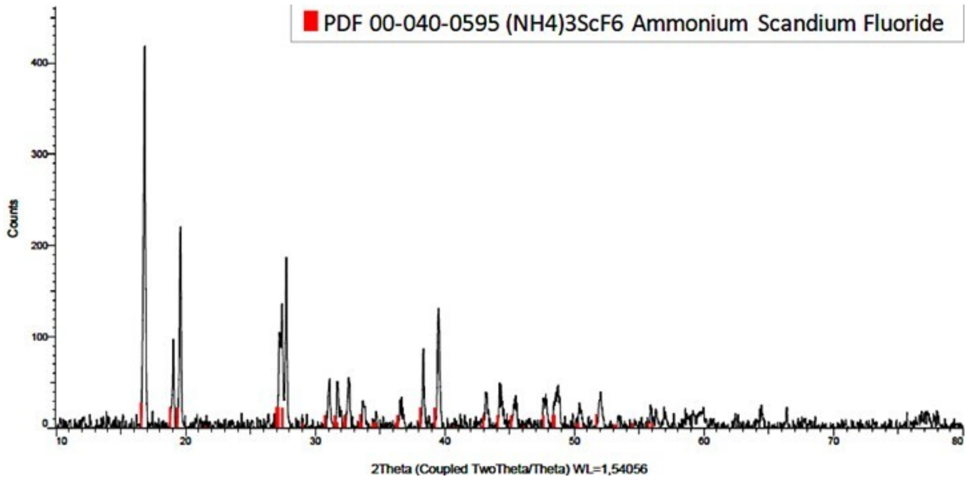

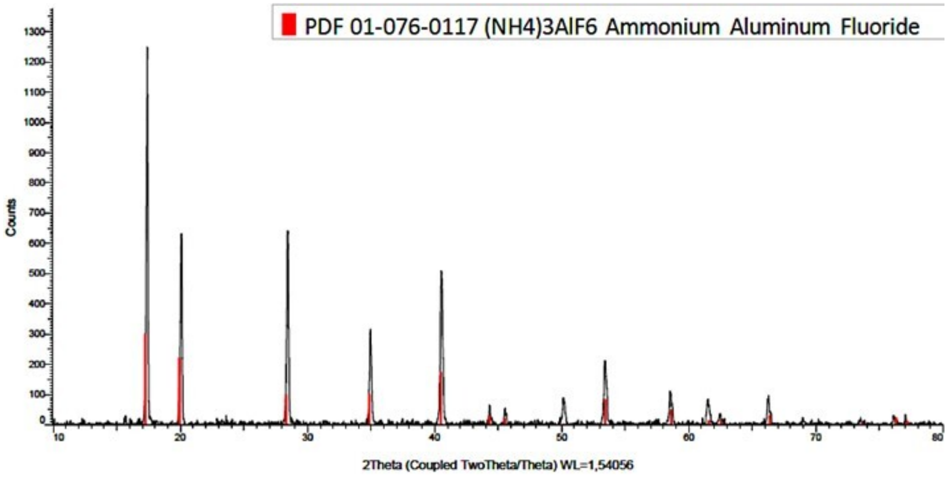

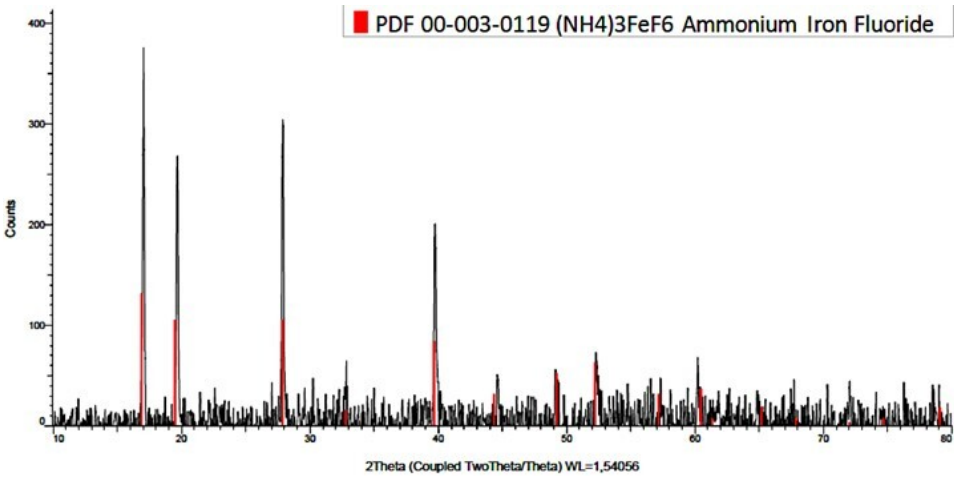

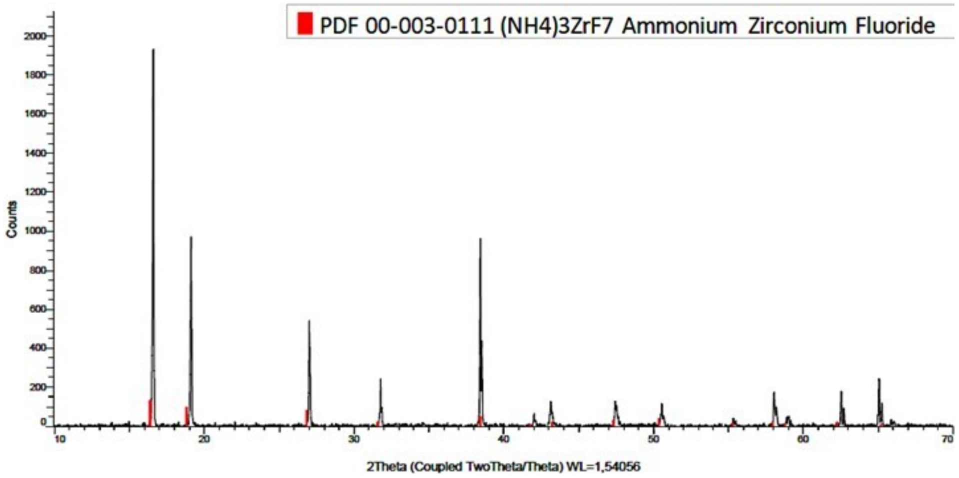

Figure S1. XRD patterns of synthesized solids (corresponds to Figure 1 in the article)

II. Volumetric charge densities of the metal ions

Table S1. Volumetric charge densities of the metal ions.

	Valency	Ionic radii (pm)	Atomic number	Electronic structure	Computed Ionic volume (pm^3)	Computed Charge density (e/pm^3)
**Zr**	4	72.0	40	[Kr] $4d^2\ 5s^2$	$1.56 \times 10^{+6}$	2.56×10^{-6}
**Sc**	3	74.5	21	[Ar] $3d^1\ 4s^2$	$1.73 \times 10^{+6}$	1.73×10^{-6}
**Fe**	3	55.0	26	[Ar] $3d^6\ 4s^2$	$6.97 \times 10^{+5}$	4.30×10^{-6}
**Al**	3	54.0	13	[Ne] $3s^2\ 3p^1$	$6.60 \times 10^{+5}$	4.55×10^{-6}
**Ti**	4	61.0	22	[Ar] $3d^2\ 4s^2$	$9.51 \times 10^{+5}$	4.21×10^{-6}

References: Lide, David R., 1995, CRC Handbook of Chemistry and Physics, 76th edition, CRC Press; Peters, E. M., 2022, Recovery of Scandium using Antisolvent Crystallization in the Valorization of Scandium-containing Waste Streams, KTH Royal Institute of Technology, Doctoral thesis

III. Phase determination of mixed solids after solubility tests in the mixed metal systems

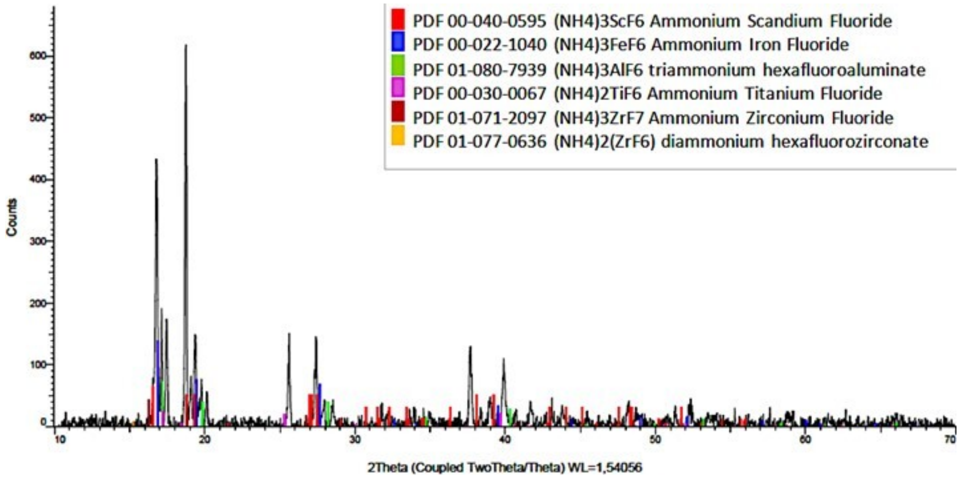

Figure S2. XRD patterns of mixed solids after solubility tests for the mixed-metal system
